# Dysexecutive symptomatology in everyday functioning and academic achievement in adolescents

**DOI:** 10.3389/fpsyg.2024.1323317

**Published:** 2024-05-28

**Authors:** María Victoria Pablo-Ríos, Enrique Navarro-Asencio, Patricia Mateos-Gordo, Raquel García-Gómez, Claudia Porras-Truque, Luis Miguel García Moreno

**Affiliations:** ^1^Faculty of Education and Psychology, Francisco de Vitoria University, Madrid, Spain; ^2^Department of Psychobiology and Methodology in Behavioral Sciences, Faculty of Education, Complutense University of Madrid, Madrid, Spain; ^3^Department of Research and Psychology in Education, Faculty of Education, Complutense University of Madrid, Madrid, Spain

**Keywords:** adolescence, language, mathematics, academic performance, dysexecutive symptomatology

## Abstract

**Background:**

During the educational stage, academic achievement depends on various social, family, and personal factors. Among the latter, executive skills in everyday life play a significant role in dealing with the academic demands of adolescents. Therefore, the aim of this study is to ascertain the effects of executive symptomatology in everyday functioning on academic achievement in adolescents.

**Method:**

The study involved 910 students aged between 13 and 15 years (M = 14.09, SD = 0.68) from both public and private schools in the Community of Madrid. The DEX, BDEFS-CA, and BRIEF-SR questionnaires were utilised to assess executive difficulties, while grades in language, mathematics, and natural sciences were used as a measure of academic achievement.

**Results:**

The data revealed statistically significant differences in working memory, emotional control, materials organisation, and task completion. In relation to language and natural sciences subjects. In the case of mathematics, emotional control and task completion were significant variables.

**Conclusion:**

Our results indicate that certain executive skills that are manifested in everyday life activities can contribute, albeit in a variable way, to academic achievement in the subjects studied. This aspect is relevant insofar as it allows us to develop preventive interventions based on the executive training of these everyday skills.

## Introduction

Every day, individuals make decisions, create plans, or choose specific behaviours in situations that allow them to lead an independent and meaningful life (Gilbert and Burgess, 2008). This is attributed to the processes involved in executive functioning or executive functions, which encompass a set of higher-level mental processes that use lower-level mental processes to activate behaviours directed towards achieving a goal (Diamond, 2013; Friedman and Miyake, 2017). These processes come into play when the task in question requires cognitive effort, as it demands additional resources compared to routine tasks, encompassing several sub-processes that contribute to the correct organisation of behaviour or decision-making. There is consensus on three basic executive functions as the basis for higher order processes such as planning, reasoning or problem solving, namely working memory (retaining information in the mind while working with it), inhibitory control (including self-monitoring and interference control) and cognitive flexibility (adapting cognitive behaviour to changing demands or priorities) (Miyake et al., 2000; Van der Sluis et al., 2007; Friedman et al., 2008; Best et al., 2011; Collins and Koechlin, 2012; Lunt et al., 2012; Diamond, 2013; Piccolo et al., 2019; Leshem and Altman, 2021; Waters et al., 2021; Friedman and Robbins, 2022; Spanou et al., 2022; Iglesias-Sarmiento et al., 2023; Xiao et al., 2024). Other models propose the distinction between “cold” and “hot” executive functions (Zelazo and Carlson, 2012), to refer, respectively, to processes involving a more cognitive and affectively neutral approach or to processes involved in motivational or emotional state. However, this distinction has not always been adequately considered (Moriguchi and Phillips, 2023) and, in research addressing this distinction and the relationship to academic performance (Poon, 2018), they found that only cold executive functions are a good predictor of academic success, while warm executive function only predicts emotional impairments. In this sense, numerous aspects of daily life require adequate executive functioning (Diamond, 2013; Cortés Pascual et al., 2019), such as controlling emotions, initiating goal-directed behaviours, or maintaining attention and ignoring distractions. However, not all sub-processes contribute equally as they depend on the demands of each situation (McAlister and Schmitter-Edgecombe, 2016).

Traditionally, executive functioning has been associated with frontal lobe activity (Exner et al., 2002; Centeno et al., 2012; Sarkis et al., 2013; Fernández García et al., 2021), specifically the prefrontal cortex (Funahashi and Andreau, 2013; Peng and Raz, 2014; Ruiz-Gutiérrez et al., 2020). However, numerous neuroimaging studies have highlighted the importance of connections with other brain regions (Stuss, 2011; Miyake and Friedman, 2012; Niendam et al., 2012). For instance, the dorsolateral prefrontal cortex has reciprocal connections with nearly all cortical and subcortical structures (Tanji and Hoshi, 2008; Yeterian et al., 2012), enabling it to integrate and control the functioning of other brain regions (Goldberg, 2001). This interconnected network plays a crucial role in an individual’s life as it is responsible for their cognitive control capacity and intellectual level (Cole et al., 2012; Rodríguez-Nieto et al., 2022).

During adolescence there is a temporary imbalance between two neurobiological systems: the subcortical socioemotional system, which alludes to emotion, rewards and novelty, and the prefrontal cognitive control system, which regulates impulse control, planning and decision-making. The socioemotional system developing more rapidly, between the ages of 14 and 17, and the cognitive control system reaches optimal development towards the end of adolescence. This is the cause of adolescents’ interest in risk-taking behaviours, seeking immediate rewards, being frustrated by delay and not measuring the consequences of their actions and behaviour on themselves and those around them (Casey et al., 2008; Steinberg, 2008).

Success associated with academic achievement plays a critical role in a student’s life and future (Kell et al., 2013). This concept refers to the knowledge and skills that determine a student’s level of productivity in a subject, which is measured through an assessment process, i.e., academic performance in a specific subject can be understood as the outcome that measures students’ achievements and knowledge acquisition, using educational methods assessed through qualitative and quantitative methods (Jiménez, 2000; Edel Navarro, 2003; Erazo, 2011; Barberá et al., 2012). For this reason, academic achievement should be considered as a multifaceted construct that encompasses different domains of learning. In our study, we considered academic performance as an indicator (product) of students’ progression and academic adaptation. For this reason, and in accordance with the indications from collaborating institutions, we have used the final califications in language, mathematics, and natural sciences as reference points. It is evident that at this educational level, these can be considered relevant factors for reflecting the academic performance of the participants. However, we understand that in order to obtain a true measure of individual academic performance, other variables should be taken into account. Different educational systems often establish objectives that include the acquisition of knowledge and skills in specific intellectual domains (e.g., mathematics, language, sciences, history), as well as in the humanities and social sciences. Therefore, academic achievement represents the level of skills and competencies that indicate the degree of achievement of specific goals that were the focus of activities conducted in educational environments, specifically in school, high school, and university settings (Steinmayr et al., 2014).

In this context, processes that develop from childhood, such as reading, verbal comprehension, numerical reasoning, and mathematical calculation, are related to executive functioning, specifically in tasks that measure cognitive flexibility, inhibitory control, and working memory. These processes lead to positive academic outcomes in children and adolescents at all school stages (Diamond and Lee, 2011; Zorza et al., 2016; Cortés Pascual et al., 2019; Romero-López et al., 2021). Moreover, in the early stages of development, executive functions appear to be more dominant, establishing a bidirectional relationship between executive functioning and academic processes such as reading comprehension (Meixner et al., 2019). Additionally, the development of working memory-related skills from a very young age is a predictive factor in the development of mathematical competence, with poor working memory potentially explaining low performance in arithmetic tasks from childhood (Aragón et al., 2019). Understanding how executive functioning influences academic skills during the crucial stage of schooling allows for its enhancement in various settings (school, family, or clinical), making it one of the best predictors of academic achievement (Ahmed et al., 2018; Fernández García et al., 2021). However, academic achievement is influenced by numerous variables that can interfere with its development, including students’ self-esteem and intrinsic motivation, which encompasses interest in learning, subject mastery, knowledge acquisition, and the perception of tasks as challenges rather than threats (Lozano et al., 2011; González-Valenzuela and Martín-Ruiz, 2019; Rodríguez-Rodríguez and Guzmán, 2019; Cid-Sillero et al., 2020). Perceived self-efficacy in the face of academic challenges (Guntern et al., 2017), teaching methods, family structure, and parents’ socioeconomic level also have a significant impact on adolescents’ academic performance (Fajardo Bullón et al., 2017).

It is indeed true that improving a student’s academic curriculum requires intervention at multiple levels. However, considering the role executive functions play in the cognitive performance needed to meet academic demands, it is advisable to understand to what extent students’ executive skills can contribute to academic achievement. The objective of this study is to determine the relationship between executive dysfunction, or executive symptomatology, in the daily lives of adolescents and their academic performance, measured through their grades in language, mathematics, and natural sciences. Therefore, it is correlational research since we try to establish if a higher executive symptomatology in adolescents is related to a worse academic achievement in the three subjects evaluated. Nevertheless, numerous studies have used these variables, and they appear to be good predictors of academic performance (Valiente-Barroso and García-García, 2012; Haapala et al., 2020; Yunus et al., 2021; Desjardins and Grandbois, 2022; Tapia-Serrano et al., 2022; de Bruijn et al., 2023). We hypothesise that adolescents showing signs of greater executive dysfunction will exhibit poorer results in language, mathematics, and natural sciences. However, the way in which this dysfunction may influence their performance could vary depending on the characteristics of their executive profile.

## Method

### Participants

A total of 933 adolescents participated (mean age 14.09 ± 0.68; range 13–15). 23 subjects were excluded either for not meeting age criteria or for not adequately completing the process, resulting in a final sample of 910 subjects (Females, 49.3%, *n* = 449; Males, 50.7%, *n* = 461). Participants were recruited from various public and private educational centres in the Community of Madrid. Both the participants and their parents or legal guardians signed an informed consent in accordance with the guidelines of the Helsinki Declaration.

Inclusion criteria required that students were enrolled in school, had provided written authorization from parents or guardians, and correctly completed the questionnaires. Exclusion criteria involved being outside the specified age range, being a second or successive student in the same academic grade (repeater student), having a disability that made it difficult to perform the tasks, having a personal history of neurological disease or a diagnosis of mental disorder. Students who received information in the classroom had to give their consent to receive documentation to take home to their parents by signing a receipt at the time they were given an envelope with the study information and informed consent. Those who did not wish to participate did not receive the documentation.

### Instruments

#### Behaviour rating inventory of executive function—self-report version (BRIEF-SR)

The BRIEF-SR, developed by Gioia, Isquith, Guy, and Kenworthy in 2000, is an executive functioning inventory composed of 80 items with three response options “never,” “sometimes” or “almost always.” It presents a Cronbach’s alpha of α = 0.96 for the global index of executive functioning, as well as favourable reliability indices on the clinical scales ranging from α = 0.75 to α = 0.96 (Guy et al., 2004; Ramírez et al., 2017; Lavigne-Cerván et al., 2021).

In this questionnaire, the individual reports various behaviours classified into eight subscales: Inhibition (analyses the ability to resist an impulse and stop one’s own behaviour at the appropriate moment), Change (measures the ability to make changes from one situation to another and to shift attentional focus), Emotional Control (assesses the affective domain of executive functions and the ability to modulate emotional responses), Initiative (measures the agility to generate ideas, responses or problem-solving strategies); Working Memory (measures the ability to hold information in the mind in order to complete a task), Planning/Organisation (determines the ability to set goals and develop appropriate steps to accomplish a task/assesses the ability to order information and extract main ideas or key concepts), Organisation of Materials (measures the ability to order, store and keep the necessary tools or instruments and to know that they are available at the time of the task), Task Completion (assesses the habits of checking and reviewing work to achieve one’s goals, as well as self-awareness of the effect of one’s behaviour on others).

In addition, it provides two main indices: Behavioural Regulation Index (measures the ability to change affect states and modulate emotions through self-control), Metacognition (analyses the ability to initiate, plan, organise and maintain in memory future-oriented problem solving) and a Global Executive Index (overall score of the eight subscales).

#### Barkley deficits in executive functioning scale—children and adolescents (BDEFS-CA)

The BDEFS-CA, developed by Barkley in 2012, is a scale that measures executive functioning. The self-administered abbreviated version consists of 20 items, in which the subject must score on a 4-point Likert-type scale ranging from “never” to “very often,” and provides two overall scores, one for the symptomatology index and one for the total score. Regarding internal consistency, it presents a high reliability index of α = 0.94 for the total score, as well as satisfactory reliability indices in all subscales ranging between α = 0.86 and α = 0.91 (Collado-Valero et al., 2021). This scale has demonstrated high correlation and validity with items measuring impulsivity, hyperactivity, and attention deficit (Collado-Valero et al., 2021; Lavigne-Cerván et al., 2021; O’Brien et al., 2021).

The BDEFS-CA scale reports on 5 areas assessed: planning (assesses the ability to understand the notion of time, as well as the ability to plan and complete tasks in order to meet deadlines), organisation (measures the organisation of one’s thoughts and actions, the ability to think quickly when unexpected events occur, find solutions and overcome obstacles that interfere with the achievement of proposed goals), self-control (assesses the ability to avoid hasty decisions, impulsive actions or comments or taking risks without thinking about the consequences), motivation (measures the level of perseverance and attitude towards certain tasks through effortfulness, finding shortcuts, the need to monitor the task or being lazy) and emotional regulation (assesses the ability to manage emotions in different situations, to remain calm and respond appropriately, as well as the use of concentration to refocus thinking towards more positive environments).

#### Dysexecutive questionnaire (DEX)

The Dysexecutive Questionnaire (DEX) is part of the Behavioural Assessment of the Dysexecutive Syndrome (BADS) battery, developed by Wilson, Alderman, Burgess, Emslie, and Evans in 1996. The DEX questionnaire has a high internal consistency (α = 0.91), making it a useful instrument for detecting dysexecutive symptomatology in the Spanish population (Pedrero-Pérez et al., 2009).

This self-administered questionnaire consists of 20 items and is scored on a 5-point Likert-type scale between “never” and “very often.” As a result it provides 5 factors: inhibition (assesses difficulties in planning, problem solving and decision making), intentionality (groups symptoms of disinhibition, euphoria and aggression), executive memory (measures symptoms of impulsivity and perseveration), positive affect (assesses symptoms of fabulation and persistence problems) and negative affect (measures lack of involvement of social rules as well as difficulties in inhibiting responses, restlessness and hyperkinesia) and a total score of the dysexecutive symptomatology.

#### Academic achievement

To determine students’ academic achievement, grades obtained in the subjects of language, mathematics, and natural sciences were used. These grades were provided, with the consent of both students and parents or guardians, by the educational institutions’ authorities at the end of the school grade, ranging from 0 to 10 points. This variable provides a true measure of their academic achievement, being a valid, representative and the most widely used measure in research addressing students’ academic performance (Valiente-Barroso and García-García, 2012; Haapala et al., 2020; Sánchez-Álvarez et al., 2020; Yunus et al., 2021; Desjardins and Grandbois, 2022; García-Gil et al., 2022; Tapia-Serrano et al., 2022; de Bruijn et al., 2023).

### Procedure

With the consent of the students and their parents or legal guardians, a series of collective sessions were conducted with 15–20 students (approximately 30–40 min) in the different schools to administer the questionnaires. These sessions lasted 2–3 days up to two weeks depending on the size of the school, took place during school hours and were supervised by the staff involved in the study and a member of the school staff. These assessments were carried out during the same academic grade, adjusting the dates according to the school’s availability. The order of application of the tests was first, the BRIEF-SR inventory, then the BDEFS-CA scale and finally the DEX questionnaire, all of them administered in paper and pencil format.

All procedures are in accordance with the Spanish legislation, Law 14/2007 of July 3, the Code of Ethical Principles for Medical Research Involving Humans Subjects outlined in the Declaration of Helsinki, and the Ethical Principles of Psychologists and Code of Conduct according to the American Psychological Association. The study was approved by the Ethics Committee of the Complutense University of Madrid (Code 19/121-E_BC).

### Statistical analysis

A non-experimental design with a correlational and predictive purpose was employed in this study (Montero and León, 2002). For statistical analysis the multivariate analysis of covariance (MANCOVA) technique was used, given the robustness of statistics even in scenarios which do not meet the conditions of supposed normality or homogeneity. Specifically, Pillai’s Trace has proven effective for the analysis of results for 2 or 3 dependent variables which do not meet the criteria for normality along with homogenous or heterogenous variances (Lateef et al., 2015; Ateş et al., 2019).

An initial verification was made to confirm the variables meet the criteria for normality and identify possible collinearity among the variables. First, we used the Kolmogorov–Smirnov test or an individualised analysis of the variables, as well as the Omnibus test of Multivariate Normality, also known as the Small’s Test, for the joint analysis of the variables. Using bivariant correlations we verified the possible collinearity between variables within each test.

Once this was confirmed, the MANCOVA was performed in several steps; first, the variables for each questionnaire were analysed to identify those with the most significant values. A joint analysis was then made of all variables which considered significant in the previous analysis. This was to avoid introducing redundant information in the MANCOVA and overloading the analysis model. As indicated, significance was evaluated using Pillai’s Trace and the effect size was determined using the partial eta squared, according to which values equal to or above 0.01 are considered small effect sizes, those equal to or above 0.06 are considered moderate and those equal to or above 0.14 are considered large (Cárdenas Castro and Arancibia Martini, 2014).

The data was analysed using the IBM Statistical Package for the Social Sciences (SPSS), version 26. The minimum confidence level used in statistical testing was 95%.

## Results

First, we will show the descriptive values for the academic variables. As shown in Table 1, the variables do not meet the criteria for normality; this is due to the slight asymmetry of the variables, with a small accumulation of cases at the high end of the curve (scores near 8, 9 and 10 points); that is, there is a lack of normality due to a greater than expected deviation at the high end. Nevertheless, the variables for performance are quantitative by nature. Table 2 specifies the descriptive statistics of the variables measuring dysexecutive symptomatology.

**Table 1:** Means, standard deviations and supposed normality for the subject’s language, mathematics, and natural sciences.

	Language	Mathematics	Natural sciences
*N*	910	910	910
Mean	6.28	5.71	6,31
Standard deviation	2.01	2.14	1.968
Asymmetry	−0.399	−0.300	−0.348
Standard Error	0.081	0.081	0.081
Kurtosis	0.124	−0.276	0.151
Standard Error	0.162	0.162	0.162
Kolmogorov–Smirnov Z	0.120	0.141	0.112
Significance (bilateral)*	0.000	0.000	0.000
Small’s test VQ3	39.9948		
Standard deviation	6.0000		
Significance*	0.0000		

a The contrast distribution is normal.

b Calculated based on the data.

*Significance for *p* < 0.05.

**Table 2:** Descriptive statistics of the variables measuring dysexecutive symptomatology.

	Mean	Standard deviation	Asymmetry	Standard error	Kurtosis	Standard error
	*(N = 910)*					
BDEFS_20_TS	35.93	9.982	0.722	0.081	0.810	0.162
BDEFS_20_SI	4.01	4.116	1.290	0.081	1.727	0.162
DEX_Inhib	8.51	5.381	0.433	0.081	−0.177	0.162
DEX_Intent	6.53	3.929	0.147	0.081	−0.505	0.162
DEX_EM	3.39	3.419	11.126	0.081	235.487	0.162
DEX_PA	3.99	3.370	7.064	0.081	124.996	0.162
DEX_NA	2.56	1.823	0.293	0.081	−0.500	0.162
DEX_Total	24.74	13.635	0.179	0.081	−0.549	0.162
BRIEF_SR In	20.34	4.808	0.628	0.081	0.180	0.162
BRIEF_SR_BC	8.42	2.008	0.421	0.081	−0.014	0.162
BRIEF_SR_CC	7.97	2.011	0.450	0.081	0.525	0.162
BRIEF_SR_TotalC	16.31	3.534	0.058	0.081	0.587	0.162
BRIEF_SR_EC	16.43	5.636	7.490	0.081	117.197	0.162
BRIEF_SR_Su	8.14	2.157	0.783	0.081	2.039	0.162
BRIEF_SR_WM	18.88	4.128	0.426	0.081	0.377	0.162
BRIEF_SR_P/O	20.72	4.378	0.187	0.081	−0.264	0.162
BRIEF_SR_MO	11.11	3.383	3.809	0.081	47.414	0.162
BRIEF_SR_TC	15.48	4.375	3.368	0.081	33.791	0.162
BRIEF_SR_BRI	62.19	24.286	13.914	0.081	261.728	0.162
BRIEF_SR_MI	65.97	13.364	0.318	0.081	0.840	0.162
BRIEF_SR_GEI	128.17	66.228	23.958	0.081	669.633	0.162

BDEFS_20_TS, BDEFS_20 Total Score; BDEFS_20_SI, BDEFS-20 Symptom Index; DEX_Inhib, DEX Inhibition; DEX_Intent, DEX Intentionality; DEX_EM, DEX Executive Memory; DEX_PA, DEX Positive Affect; DEX_NA, DEX Negative Affect; DEX_Total, DEX Total; BRIEF_SR_IN, BRIEF_SR Inhibition; BRIEF_SR_BC, BRIEF_SR Behavioural Change; BRIEF_SR_CC, BRIEF_SR Cognitive Change; BRIEF_SR_TotalC, BRIEF_SR Total Change; BRIEF_SR_EC, BRIEF_SR Emotional Control; BRIEF_SR_Su, BRIEF_SR Supervision; BRIEF_SR_WM, BRIEF_SR Working Memory; BRIEF_SR_P/O, BRIEF_SR Planning/Organisation; BRIEF_SR_MO, BRIEF_SR Materials Organisation; BRIEF_SR_TC, BRIEF_SR Task Completion; BRIEF_SR_BRI, BRIEF_SR Behavioural Regulation Index; BRIEF_SR_MI, BRIEF_SR Metacognitive Index; BRIEF_SR_GEI, BRIEF_SR Global Executive Index.

As a result of the collinearity analysis, certain variables were removed as they provided similar parameters. Therefore, the decision was made to exclude the total scores and analyse the effect of each dimension on the scores of individual subjects (Table 3).

**Table 3:** List of eliminated variables.

Questionnaire	Eliminated variable	Pearson’s correlation	Retained variable
BDEFS-20	Total score	0.877	Index of symptoms
DEX	Total score	0.904	Inhibition
		0.867	Intentionality
BRIEF-SR	Total change	0.820	Behavioural change
		0.820	Cognitive change
	Meta-cognitive index	0.855	Working memory
		0.840	Planning / Organisation

*Significance for *p* < 0.01.

The general variables with a collinearity equal to or higher than 0.8 were eliminated.

A MANCOVA was then carried out which showed statistically significant differences in academic performance of students, although with small effect size (Table 4). The rest of the variables of the BRIEF-SR, and those of the DEX, did not show any significant relation to the dependent variable. Table 5, shows the impact of the variables of executive functioning for the three subjects, indicating how students with greater executive dysfunction showed poorer academic achievement. Overall, variables such as Symptom Index (BDEFS-20), Intentionality (DEX), Inhibition, Working Memory, and Task Completion (BRIEF-SR) showed significant differences across all three subjects.

**Table 4:** Results of the multivariate analysis of covariance (MANCOVA) by questionnaire.

Variable	Pillai’s Trace (V)	*F*	Gl of hypothesis	Gl of error	p^*^	Partial ƞ^2^
BDEFS_20_SI	0.055	17.530^a^	3	906	0.000	0.055
DEX_Inhib	0.013	3.971^a^	3	902	0.008	0.013
DEX_Intent	0.025	7.678^a^	3	902	0.000	0.025
BRIEF_SR_IN	0.015	4.427^a^	3	902	0.004	0.015
BRIEF_SR_EC	0.010	3.017^a^	3	902	0.029	0.010
BRIEF_SR_WM	0.015	4.516^a^	3	902	0.004	0.015
BRIEF_SR_MO	0.010	3.156^a^	3	902	0.024	0.010
BRIEF_SR_TC	0.024	7.471^a^	3	902	0.000	0.024

BDEFS_20_SI, BDEFS-20 Symptom Index; DEX_Inhib, DEX Inhibition; DEX_Intent, DEX Intentionality; BRIEF_SR_IN, BRIEF_SR Inhibition; BRIEF_SR_EC, BRIEF_SR Emotional Control; BRIEF_SR_MO, BRIEF_SR Materials Organisation; BRIEF_SR_TC, BRIEF_SR Task Completion.

*Significance for *p* < 0.05.

a Exact statistic.

**Table 5:** Results for BDEFS-20, DEX and BRIEF-SR variables for language, mathematics, and natural sciences.

Variables / Subjects	Language					Mathematics					Natural sciences				
				*β*					*β*					*β*	
	*F*	p^*^	Partial ƞ^2^	Intersection	Variable	*F*	p^*^	Partial ƞ^2^	Intersection	Variable	*F*	p^*^	Partial ƞ^2^	Intersection	Variable
BDEFS_20_IS	37.050	0.000	0.039	6.668	−0.097	38.484	0.000	0.041	6.128	−0.105	46.411	0.000	0.049	6.728	−0.105
DEX_Inhib						4.875	0.028	0.005	6.560	−0.044	10.935	0.001	0.012	7.123	−0.060
DEX_Intent	22.839	0.000	0.025	7.032	−0.124	11.399	0.001	0.012	6.560	−0.094	9.295	0.002	0.010	7.123	−0.077
BRIEF_SR_IN	4.802	0.029	0.005	8.665	−0.039	5.203	0.023	0.006	8.459	−0.044	13.183	0.000	0.014	9.169	−0.063
BRIEF_SR_EC	7.689	0.006	0.008	8.665	0.036						6.794	0.009	0.007	9.169	0.033
BRIEF_SR_WM	10.005	0.002	0.011	8.665	−0.071	7.597	0.006	0.008	8.459	−0.066	12.390	0.000	0.014	9.169	−0.076
BRIEF_SR_MO	5.277	0.022	0.006	8.665	0.057										
BRIEF_SR_TC	21.241	0.000	0.023	8.665	−0.096	10.110	0.002	0.011	8.459	−0.071	15.248	0.000	0.017	9.169	−0.078

BDEFS_20_IS, BDEFS-20 Symptom Index; DEX_Inhib, DEX Inhibition; DEX_Intent, DEX Intentionality; BRIEF_SR_IN, BRIEF_SR Inhibition; BRIEF_SR_EC, BRIEF_SR Emotional Control; BRIEF_SR_MO, BRIEF_SR Materials Organisation; BRIEF_SR_TC, BRIEF_SR Task Completion.

*Significance for *p* < 0.05.

Table 6 shows the results of the MANCOVA of relevant, jointly analysed variables. The results show that the variables of the BRIEF (except for the variable Inhibition) are the most determinant, while the variables of the BDEFS-20 and of the DEX are represented by these and therefore considered not significant.

**Table 6:** Results of the multivariate analysis of covariance (MANCOVA) for relevant variables.

Variable	Pillai’s Trace (V)	*F*	Gl of hypothesis	Gl of error	p^*^	Partial ƞ^2^
BRIEF_SR_EC	0.012	3.576^a^	3	899	0.014	0.012
BRIEF_SR_WM	0.009	2.822	3	899	0.038	0.009
BRIEF_SR_MO	0.011	3.300^a^	3	899	0.020	0.011
BRIEF_SR_TC	0.020	6.037^a^	3	899	0.000	0.020

BRIEF_SR_EC, BRIEF_SR Emotional Control; BRIEF_SR_WM, BRIEF_SR working memory; BRIEF_SR_MO, BRIEF_SR Materials Organisation; BRIEF_SR_TC, BRIEF_SR Task Completion.

*Significación para *p* < 0.05.

a Estadístico exacto.

Finally, Table 7 shows the results of the analysis of the most relevant variables by subject. For the subject *language*, four variables of the BRIEF were significant: “emotional control” and “organisation of materials” showed significant differences (positive) and the variables “working memory” and “task completion” showed significant differences (negative). These results are in line with those obtained in the analysis of the questionnaires (Table 5). Regarding the subject *mathematics*, the variable “emotional control”, which was not significant in the previous analysis, showed significant differences in the positive sense, with performance in mathematics; the variable “task completion” showed significant differences (negative) with results similar to the previous analysis (Table 5). In turn, the variables “working memory” and “organisation of materials” were not significant in this analysis. For the subject *natural sciences*, the variable “organisation of materials”, which was not significant in the previous analysis did show significant differences (positive) with the dependent variable; the rest of the variables coincided with the previous analysis (Table 5), with significant differences for “emotional control” (positive) and “working memory” and “task completion” (negative).

**Table 7:** Results for the relevant variables of BRIEF-SR for the subject’s language, mathematics, and natural sciences.

Variables / Subjects	Language			*β*		Mathematics			*β*		Natural sciences			*β*	
	F	*p*	Partial ƞ^2^	Intersection	Variable	*F*	*p*	Partial ƞ^2^	Intersection	Variable	*F*	*p*	Partial ƞ^2^	Intersection	Variable
BRIEF_SR_EC	9.684	0.002	0.011	8.103	0.041	3.656	0.056	0.004	7.933	0.027	7.538	0.006	0.008	8.840	0.035
BRIEF_SR_WM	3.959	0.047	0.004	8.103	−0.048						8.483	0.004	0.009	8.840	−0.068
BRIEF_SR_MO	5.822	0.016	0.006	8.103	0.059						4.027	0.045	0.004	8.840	0.048
BRIEF_SR_TC	16.473	0.000	0.018	8.103	−0.086	7.671	0.006	0.008	7.933	−0.063	13.375	0.000	0.015	8.840	−0.075

BRIEF_SR_EC, BRIEF_SR Emotional Control; BRIEF_SR_WM, BRIEF_SR Working Memory; BRIEF_SR_MO, BRIEF_SR Materials Organisation; BRIEF_SR_TC, BRIEF_SR Task Completion.

*Significación para *p* < 0.05.

a Estadístico exacto.

## Discussion

The principal objective of this research was to assess the relation between dysexecutive symptomatology among adolescents and their academic performance in the subjects of language, mathematics, and natural sciences. In general terms, the results showed that students with a higher degree of dysexecutive symptoms showed poorer results. Difficulties in impulse control, working memory and cognitive flexibility are related to worse calcifications in the three subjects evaluated. It is necessary to point that the three core executive functions and the high order executive functions (Diamond, 2013) are processes clearly involved in most daily activities and that, the difficulties in these activities, is which we have evaluated to determine the executive profile of the individuals. That means that when an individual scores high in, for example, the variable “task completion,” we can deduce that he has difficulties in executive functions like working memory, inhibitory control or planning, processes involved in the mentioned task. Furthermore, it should be noted that the dysfunctional dysfunction observed in students can be considered a dysregulatory behaviour of the subject him/herself (procrastinating tasks, limiting his/her effort, etc.), but also the consequence of the context in which he/she is immersed, as an under-stimulating educational experience contributes to the loss of interest in learning and the need to focus attention on other, more attractive tasks (de la Fuente Arias, 2017; de la Fuente et al., 2022).

In the subject of language, for example, students with greater dysexecutive symptoms experienced more difficulties in handling different types of information at the same time, in continuing a task after a certain period, managing their tasks, and detecting their possible errors. Given that reading comprehension is a strong predictor of academic performance among adolescents and a key element in the acquisition of knowledge (González-Valenzuela and Martín-Ruiz, 2019), difficulties in cognitive flexibility and working memory have a direct impact on reading ability (Latzman et al., 2010; Leshem and Altman, 2021). Similarly, the relation between working memory and reading comprehension involves the student’s ability to retain information (in their memory) while integrating this is higher processes; thus, difficulties or impairment of working memory is directly associated with diminished reading comprehension skills (Stelzer and Cervigni, 2011). In Spanish educational regulations, the competences to be acquired in the subject of language, at the educational stage where we recruited the sample, depend largely on reading comprehension (Caballero-Cobos and Llorent, 2022). Furthermore, the relation between working memory and impaired reading comprehension is particularly important for young people. A coherent mental representation of a text requires that inhibitory processes disregard or ignore irrelevant information, while working memory processes and stores information for the mental construction of meaning of the text. This process is consolidated over time and adolescents experiencing difficulties in these functions are therefore particularly vulnerable in terms of their future skills development (Demagistri et al., 2014).

Achievement in mathematics is enhanced by greater cognitive flexibility, alternating attention, cognitive processing speed and working memory (Valiente-Barroso and García-García, 2012; Allen and Giofrè, 2021), as well as processes related to emotional regulation (Kahl et al., 2021). Regarding working memory, there is a great deal of scientific evidence that good achievement in mathematics, especially among children in primary education, although in our study this variable was significant in the initial results but ceased to show significant differences when analysing only the variables of the BRIEF-SR. This may be due to the fact that, firstly, to the maturity of neural systems in adolescents, since greater functional specialisation over time leads to a reduction in the use of working memory and attentional resources (Rivera et al., 2005; Gilmore et al., 2020); secondly, it has been shown that the development of effective strategies of emotional control, a significant variable in our study, helps to enhance working memory even when performance is poor (Kahl et al., 2021). These findings are in line with those of other studies which found that students with low attentional capacity, poor concentration and high levels of impulsivity and behaviour problems show poorer learning outcomes in mathematics (Suárez-Riveiro et al., 2020). This is characterised by difficulties in breaking down problems into shorter steps, alternative operations when working with numbers and symbols, and problems in disregarding irrelevant or redundant information in problem solving (Bull and Lee, 2014).

In order to perform well in mathematics, adolescents need certain cognitive skills such as working memory, impulse control, cognitive flexibility, attention and processing speed, among others (Valiente-Barroso and García-García, 2012), the first two being the most important predictors since preschool (Monette et al., 2011; Presentación-Herrero et al., 2015; Allen and Giofrè, 2021); academic performance in mathematics is also linked to good emotional regulation (Kahl et al., 2021). In this way, working memory is in constant action, updating cognitive load as it integrates data while successfully executing the desired behaviour; Inhibitory control, in turn, acts as a filter, discarding unnecessary information and facilitating strategy change for effective planning (Resing et al., 2019; Gilmore et al., 2020).

With respect to the subject of natural sciences, we observed that adolescents with difficulties in tasks that require cognitive flexibility obtain lower academic performance. This may be because this subject, like some processes in language, such as reading, requires more changes in mental schemas, the initiation of problem-solving behaviour and the formulation of new concepts (Latzman et al., 2010). Furthermore, as the difficulties in processing using executive functions increases, behavioural problems are increased, especially impulsiveness, hyperactivity, and attention deficits along with a lack of awareness of the consequences of one’s actions (Ramos-Galarza et al., 2019).

The study found that cognitive skills are closely associated with academic achievement, a relationship that appearing in early childhood and reaching a critical juncture in early adolescence (Jacobson et al., 2011; Ahmed et al., 2018), although this relation varies depending on the executive functions involved and the type of tasks used to measure performance within each subject. Our research showed that working memory and cognitive flexibility are associated with reading comprehension and mathematical ability, while impulse control is associated principally with the latter; thus, it can be affirmed that some executive functions are more predictive than others (Waters et al., 2021). Nevertheless, several studies have questioned the weight of executive functions in academic performance, including the study by Dubuc et al. (2020) which found that neither impulse control nor working memory are relevant predictors of academic performance in mathematics and science when compared to other variables. Executive functioning are a complex structure and are, above all, ever-changing (Best et al., 2011); they may develop differently depending on the maturity of the individual and those related to emotional processing or more closely associated with motivation tend to mature more slowly than those without these associations (Peng and Kievit, 2020); this suggests that emotional regulation is also a good predictor of academic success (Vinter et al., 2021).

It should be noted, however, that academic achievement is measured by other variables which partly explains the differences in learning outcomes and in cognitive functioning itself. For example, studies using neuroimaging suggest that the socioeconomic status of students is associated with structural and functional differences in the hippocampus, the amygdala, and the prefrontal cortex (Ahmed et al., 2018; Piccolo et al., 2019), structures which can have a direct or indirect influence on cognitive functioning. Furthermore, the intrinsic motivation of the student, the desire to learn and perceived self-efficacy are also good predictors of academic performance (Fajardo Bullón et al., 2017; Guntern et al., 2017; González-Valenzuela and Martín-Ruiz, 2019); contrarily, high levels of stress negatively affect the executive functions related to attention, planning, organisation of tasks, among others, hindering the academic performance of students (Suárez-Riveiro et al., 2020).

The present study has several limitations that should be taken into account for future research. To begin with, although the sample size was considerable, the results did not show a normal distribution, as many scores were in the 3 to 4 and 6 to 8 range, which could introduce skewness into the results. In addition, the use of final numerical grades for each subject does not represent the most reliable measure of student performance; this may introduce some bias in determining the actual achievement of students. Also, the procedure for arriving at this final grade may vary between schools. Another aspect to consider in future studies is the fact that the selected sample consisted of students from two different grades, which may be a factor of variability that has not been adequately controlled for. The limitations imposed by the collaborating centres and the restrictions established by the law on data protection for minors have conditioned, in part, the possibility of carrying out a series of more specific measures to optimise data collection. Finally, this research has focused from a molecular perspective, i.e., it focuses on the action of the student’s cognitive functioning on their academic achievement, leaving aside a molar perspective that refers to the influence that teaching and assessment processes can have on the educational context in which the student moves (de la Fuente et al., 2019), which is why it would be advisable to integrate both levels. For future research, it would be ideal to use a more homogeneous sample in order to be able to compare the results more effectively, as well as to evaluate academic achievement in the subjects of language, mathematics and natural sciences using a specific test designed for the research that, together with the final average mark for each subject, would provide us with relevant information on student performance. Finally, it would be useful to conduct follow-up interviews and questionnaires to assess whether these results persist over time or evolve as adolescents mature, as well as to investigate possible differences related to gender and age.

In conclusion, and considering the limitations mentioned above, the results of this research contribute to determining the impact of executive functioning on the learning processes and academic achievement of the students. From our perspective, we consider it necessary to create a training programme that includes strategies to regulate impulsivity and the immediate search for rewards, the development of skills that allow greater mastery of emotions and the increase of skills related to student planning and organisation that allow the student to obtain satisfactory academic results. It is hoped that these findings can serve to lay the foundations for the development of intervention strategies and protocols that can be applied in the early stages of education.

## Data availability statement

The original contributions presented in the study are included in the article, further inquiries can be directed to the corresponding author.

## Ethics statement

All procedures are in accordance with the Spanish legislation, Law 14/2007 of July 3, the Code of Ethical Principles for Medical Research Involving Humans Subjects outlined in the Declaration of Helsinki, and the Ethical Principles of Psychologists and Code of Conduct according to the American Psychological Association. The study was approved by the Ethics Committee of the Complutense University of Madrid (Code 19/121-E_BC). Both the participants and their parents or legal guardians signed an informed consent in accordance with the guidelines of the Helsinki Declaration.

## Author contributions

MP-R: Writing – original draft, Data curation, Formal analysis, Investigation, Methodology, Writing – review & editing. EA: Data curation, Formal analysis, Methodology, Software, Writing – original draft. PG: Data curation, Investigation, Resources, Writing – original draft. RG-G: Data curation, Investigation, Resources, Writing – original draft. CT: Data curation, Investigation, Resources, Writing – original draft. LG: Conceptualization, Funding acquisition, Resources, Supervision, Writing – original draft, Writing – review & editing.
